# The p-i Concept: Pharmacological Interaction of Drugs With Immune Receptors

**DOI:** 10.1097/WOX.0b013e3181778282

**Published:** 2008-06-15

**Authors:** Werner J Pichler

**Affiliations:** 1 Division of Allergology, Clinic for Rheumatology and Clinical Immunology/Allergology, University of Berne, Berne, Switzerland

**Keywords:** p-i concept, drug hypersensitivity, hapten, prohapten, T-cell receptor, T cells

## Abstract

The immune response in drug hypersensitivity is normally explained by the hapten hypothesis. It postulates that drugs with a molecular weight of less than 1000 D are too small to cause an immune response per se. However, if a chemically reactive drug or drug metabolite binds covalently to a protein and thus forms a so-called hapten-carrier complex, this modified protein can induce an immune response. This concept has recently been supplemented by the p-i concept (or pharmacological interaction with immune receptors), which postulates that some drugs that lack hapten characteristics can bind directly and reversibly (noncovalently) to immune receptors and thereby stimulate the cells. For example, a certain drug may bind to a particular T-cell receptor, and this binding suffices to stimulate the T cell to secrete cytokines, to proliferate, and to exert cytotoxicity. The p-i concept has major implications for our understanding of drug interaction with the specific immune system and for drug hypersensitivity reactions. It is based on extensive investigations of T-cell clones reacting with the drug and recently of hybridoma cells transfected with the drug-specific T-cell receptor for antigen (TCR). It is a highly specific interaction dependent on the expression of a TCR into which the drug can bind with sufficient affinity to cause signaling. Small modification of the drug structure may already abrogate reactivity. Stimulation of T cells occurs within minutes as revealed by rapid Ca^++ ^influx after drug addition to drug-specific T-cell clones or hybridoma cells, thus, before metabolism and processing can occur. As the immune system can only react in an immunologic way, the symptoms arising after drug stimulation of immune receptors imitate an immune response after recognition of a peptide antigen, although it is actually a pharmacological stimulation of some T cells via their TCRs. Clinically, the p-i concept could explain the sometimes rapid appearance of symptoms without previous sensitizations and the sometimes chaotic immune reaction of drug hypersensitivity with participation of different immune mechanisms while normal immune reactions to antigens are highly coordinated. Nevertheless, because the reactions lead to expansion of drug-reactive cells, many features such as skin test reactivity and stronger reactivity upon reexposure are identical to real immune reactions.

## 

In drug hypersensitivity, 2 extremely polymorphic systems encounter each other. On one hand, modern chemistry, which allows synthesis of an endless amount of drugs often designed to fit into and thus blocking receptors or enzymes and, on the other hand, the specific immune system, which is composed of T and B cells expressing greater than 10^9 ^different T-or B-cell receptors for antigen (TCR, BCR). The enormous polymorphism of the immune system allows it to react to all kinds of different antigens and even to antigens that will be created in the future. It surely does also express receptors into which some old or new drugs may fit. On the other hand, it is an immunologic dogma that small molecules do not stimulate the immune system. However, is this so? Or are there exceptions to this rule?

## The Hapten and Prohapten Concept

An immune reaction starts by involvement of the innate immune system. The antigen (bacterium, virus, etc) stimulates the innate immune system via, for example, Toll-like receptors (TLRs) on dendritic cells and thereby sets an initial alarm signal. The activated dendritic cells function as antigen-presenting cells (APCs) as they take up and process complex antigens, which subsequently are presented as peptides to T cells in a suitable environment, mainly the lymph nodes. The ensuing immune response is variable and efficient, as different antigens such as soluble or cell-bound viral antigens elicit a distinct immune response, capable of eliminating the infectious agent.

Small low-molecular weight compounds (< 1000 D) are thought to be too small to elicit such an immune response per se. Nevertheless, small compounds such as drugs or metal ions were found to be able to trigger an immune response [1-5]. The hapten (and prohapten) model was developed and is currently the accepted explanation for these observations: Chemically reactive, small compounds (haptens) bind to proteins or peptides and modify them [4-6]. These haptens have thereby 2 essential features for inducing an immune response.

**Figure 1:**
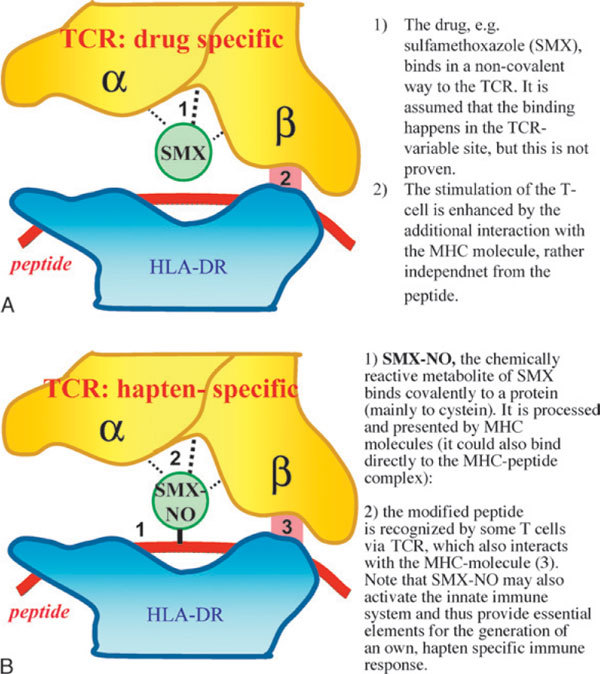
**The p-i concept**. A schematic representation of the p-i concept in comparison with the hapten concept. A, In the p-i concept, the drug (eg, SMX) binds to the TCR and provides some initial signal. In most instances, this signal is insufficient to induce T-cell activation with cytokine synthesis and proliferation. The signal is strengthened by the additional interaction with HLA molecules. It seems to be rather independent of the type of peptide embedded and sometimes even from the HLA allele. Drug binding and HLA interactions stimulate T cells like a normal peptide/HLA complex. Because this way of T-cell stimulation does not follow the rules of the development of a normal immune response, the subsequent activations appear clinically and upon in vitro analysis often chaotic and uncoordinated. B, In the hapten hypothesis, the hapten-modified peptide is recognized and stimulates T cells. The hapten may also have the ability to activate the innate immune system, for example, dendritic cells.

a) They may stimulate the innate immune system by covalently binding to cellular proteins and thereby transmit a danger signal, which results in stimulation of cells of the innate immune system,[7-9] such as up-regulation of CD40. Exceptionally, they may happen to bind to TLR7 and TLR8 directly and thus stimulate dendritic cells. This was shown for imiquimod [10].

b) They may stimulate the specific immune system. By forming hapten-carrier complexes, they form neoantigenic structures. This hapten-protein complex is processed and presented as hapten-modified peptide to T cells, which can react with it [1-4,11]. Modified proteins can also be immunogenic for B cells and thus elicit a humoral immune response. This hapten modification can occur with soluble proteins, cell-bound proteins, or with the major histocompatibility complex (MHC)-peptide complexes themselves, leading to distinct immune responses (Figure 1) [1,3].

Prohaptens are per se not chemically reactive and thus unable to form a covalent bridge to peptides. To become chemically reactive, they first need to be converted into a hapten by being metabolized into a compound that is chemically reactive [1,11-14]. Metabolism may occur in the liver, where it may not necessarily induce an immune response, but actual tolerance [15]. On the other hand, if a reactive compound escapes the tolerogenic environment of the liver or if the immune response to the compound is developed in lymph nodes outside of the liver (such as probably in severe drug hypersensitivity syndromes also called drug rash with eosinophilia and systemic symptoms), a systemic reaction mainly affecting the skin and with an accompanying hepatitis may occur [16,17].

## The p-i Concept

The hapten and prohapten concept elegantly circumvents the (presumed) blindness of the immune system for low-molecular weight compounds by postulating chemical reactivity and subsequent coupling to a macromolecular carrier an absolute necessity. As a consequence, drugs and other substances that are incapable of such conjugation with a carrier would not be antigens and could not induce hypersensitivity reactions.

We have recently challenged this dogma and proposed a third model, which is meant to supplement the hapten/prohapten concept [1,3,18]. Termed the p-i concept, which stands for "direct pharmacological interaction of drugs with immune receptors," it states that certain drugs would bind specifically and reversibly to some of the highly variable antigen-specific receptors in a direct way, instead of covalently modifying the MHC-peptide complex, which are the 2 feasible "partners" to accommodate allergy-inducing drugs. Such a drug-TCR interaction would be metabolism and processing independent and in fact mimics drug interactions with other nonimmunologic receptors. Although the MHC-peptide complex would not contribute (much) to the binding energy, it would still be necessary (1) for full T-cell activation and (2) to direct the cytotoxic immune response to a target cell. This model has been elucidated for TCR, but it is possible that BCR on B cells are activated via a similar mechanism.

**Table 1:** The p-i Concept-Pharmacological Interaction With Immune Receptors

A chemically inert drug, unable to covalently bind to proteins, is still able to fit into some of the many immune receptors (as it does into other proteins/receptors). This reversible drug-receptor interaction can, under certain circumstances, activate immune cells specific for peptide antigens, which expand and can cause inflammatory reactions of different types. Such a reaction would not need the generation of one's own immune response to the drug, albeit an expansion of drug-reactive cells may be required before symptoms appear.

### Immunologic Data using Drug-Reactive T-Cell Clones

This p-i concept was developed over the last 10 years in our group in Bern and relies on immunologic and clinical findings. The generation of drug-reactive (so-called drug-specific) T-cell clones (TCCs) allowed better analysis of drug-T cell interactions and subsequent stimulations. Several observations argue against processing or covalent binding and favor the p-i concept.

1. Only some T cells of a patient with drug hypersensitivity react with the drug. Using TCC, many different chemically inert drugs were found to be able to stimulate T cells via the TCR in an MHC-dependent way, in particular, lamotrigine,[12] carbamazepine,[13] sulfamethoxazole (SMX),[19] mepivacaine and lidocaine,[20]*p*-phenylenediamine,[21] and ciprofloxacin or moxifloxacin [22,23].

2. Specific TCC reacted even if the APCs were fixed by glutaraldehyde, excluding that either processing or intracellular metabolism is involved [14,19,20].

3. Upon pulsing of APCs (incubation of APCs with the drug for 1 hour followed by 2 washing steps), no stimulation of drug-specific T-cell clones was observed for lidocaine, lamotrigine, carbamazepine, ciprofloxacin, and SMX[14,19,21,22] as the washing removed the labile bound drug. Sulfamethoxazole has been characterized particularly well because the reactive metabolite of SMX (SMX-nitroso [SMX-NO]) was available for comparison [24,25]. Sulfamethoxazole-nitroso acts as a typical hapten, capable of covalently modifying the MHC-peptide complex. This hapten binding results in a very stable binding, which cannot be washed away and was still able to stimulate hapten-reactive T cells after washing [19].

4. For a number of drugs, the kinetics of T-cell activation are simply much too fast for any involvement of antigen processing. In the presence of APCs, lidocaine and SMX activate T cells quasi immediately, as revealed by a rapid (within 1 minute) and sustained intracellular _Ca_^2+ ^increase [19]. It is impossible to reconcile this timing with an intermediate metabolism and processing step, which needs 60 minutes or longer to occur. In addition, the kinetics of TCR down-regulation on drug-reactive TCC after encountering the inert drug are similar to the recognition of preprocessed immunogenic peptides (occurring within the first 30 minutes) and clearly differ from the recognition of proteins, which requires several hours [19].

5. Hypersensitivity to the drug SMX (Figure 1A) was thought to be a consequence of bioactivation to the hydroxylamine metabolite (SMX-NHOH) and further oxidation to the ultimate reactive metabolite SMX-NO. The antioxidant glutathione is known to protect cells from reactive metabolites such as SMX-NO by conjugation and subsequent dissociation to SMX-NHOH and/or SMX [26]. Addition of glutathione to peripheral blood mononuclear cells enhanced rather than reduced the proliferation to SMX metabolites, presumably by transforming SMX-NO back to the "original" antigen, SMX [26]. The response of SMX-NO-specific TCC was abrogated when glutathione was present during the covalent modification of APCs. Collectively, these experiments support the concept that T cells in allergic individuals recognize the noncovalently bound parent drug SMX rather than APC covalently modified by SMX-NO.

6. Drug-specific TCCs show some peculiar features reminiscent of superantigen stimulations but not seen with classic peptide antigens. Many drug-specific TCCs were found to be MHC unrestricted,[27] and the frequency of alloreactivity of the drug-reactive TCC is much higher among drug-than peptide-specific TCCs from the same donor [28]. In other words, about 30% of drug-specific TCCs did react with an alloantigen without drug [28]. The MHC-bound peptide seems to be irrelevant for SMX-specific T-cell activation [29]. Lastly, drugs simultaneously elicit a CD4 and CD8 T-cell response to the same compound. These are features observed with superantigens such as SEB (staphylococcal euterotoxin B) [18].

7. The generation of one's own immune response (often involving T and B cells) happens with hapten(-carrier) constructs, but not if the cells are stimulated by the drug via the p-i concept. In the p-i concept, there is a remarkable dissociation between T-cell and B-cell responses: in the better studied T-cell stimulations, often exclusively T cells react, but no B cells. It is assumed that no sensitization phase-as it is typical for an immune response-occurs: if enough cells are able to react with the drug, symptoms may arise quite rapidly at the first encounter. On the other hand, if only a few T cells react with the drug, a time interval of a few days may be observed, similar to an intermittent sensitization phase in immune responses. Actually, it is assumed--but not yet proven--that the p-i-stimulated T cells are memory T cells (with an additional peptide specificity), as the memory T cells may have a higher readiness to react to such a minor signal as drug binding to TCR [30]. Moreover, this level of reactivity may be even more reduced by a concomitant virus infection. This would explain the higher incidence of drug allergies in systemic virus infections by herpes viruses or human immunodeficiency virus (HIV).

8. In the p-i concept, pharmacology meets immunology. This raises some semantic problems because each of these 2 areas uses its special words to express its findings: for example, in immunology, where only one of million similar receptors react with a particular antigen, one calls it *recognition of an antigen*, whereas in pharmacology, a basically similar interaction is called *ligand binding *to its receptor and not called *recognition*. Similarly, the term *specific *for an antigen has its particular meaning in immunology, where affinity maturation is one important concept and somehow implies an active process. Consequently, one speaks of peptide-specific T-cell clones and so on as one particular receptor binds a particular antigen while another, almost identical, receptor binds to another antigen. In pharmacology, this enormous polymorphism of functionally identical receptors is lacking, and normally only one or a few receptors have one or a few natural ligands. Is the TCR-*specific for a certain peptide*-which happened to be also stimulated by a drug, now *specific *for the drug, or is the drug binding just an ("accidental") additional interaction? *Specificity *seems to be the wrong term for this pharmacological interaction with an immune receptor. These semantic problems seem trivial, but can be a constant reason for misunderstandings.

### Immunological Data using T-Cell Receptor-Transfected Hybridoma Cells

To further study these unusual characteristics of drug-T cell interactions, we recently developed drug-specific TCR transfectants. It unequivocally demonstrated that T-cell activation by drugs is TCR dependent [23,31]. Four SMX-specific, 2 quinolones, and 2 contrast media-reactive human TCR were introduced into the mouse T-cell hybridoma cell line 54ζ17 (O. Acuto, Paris, France) according to the method described by Vollmer et al.[32] These transfectants expressed drug-specific TCR on the cell surface and could be stimulated by the relevant drug in a specific way in the presence of APCs, resulting in interleukin 2 secretion. These TCR-transfected hybridoma cells behaved like drug-specific TCC, as the drug could be washed away (contrary to haptens covalently bound to carrier molecules), the presence of APC (MHC) was required for interleukin 2 production, and fixed APC were still able to present the drug. Similarly, the kinetics of TCR activation were too fast to involve antigen processing, as antigen-dependent extracellular signal-regulated kinase (ERK) phosphorylation was detected within 1 minute of SMX exposure [31]. Shuffling of different, SMX-or quinolone-specific TCR α or β chains revealed that recognition depends on the coordinated expression of both original TCR chains to keep reactivity to the drugs [23].

A further support for the p-i concept comes from the recent elegant study by Chen's group from Taiwan [33,34]. After having demonstrated the high association of Stevens-Johnson syndrome to HLA-B*1502 in Han Chinese, they eluted the peptides from HLA-B*1502-positive cell lines incubated with carbamazepine. The eluated peptides did not contain any covalently bound carbamazepine, and the authors conclude that their data would support the p-i concept [34].

### The p-i Concept: Bypassing the Innate Immune System Because of Cross-Reactivity with Peptide Antigens

The p-i concept has major implications for our interpretation of drug-induced immune-mediated side effects. It actually puts some drug-induced hypersensitivity reactions outside the rules for a normal immune response [35]. This could explain some peculiar clinical findings, namely that drug-induced T cell-mediated skin reactions can occur already within a few hours after administration and/or without previous exposure to the drug, as for example, documented for radio-contrast media [36]. The kinetics of such a reaction are much too fast to be explained by the induction of a classic primary response, which is mounted in the course of several days only.

To explain these phenomena, we proposed that the drugs are bypassing the innate immune system as they stimulate memory T cells (with a peptide specificity) [30]. Such memory T cells have a lower threshold of reactivity compared with naive T cells, which might even be further decreased if a generalized immune reaction with its abundance of cytokines is occurring. A secondary memory response by the immune system is generally much faster and can lead to an immune reaction within the time frame observed for some adverse drug reactions. Moreover, these features would explain the higher incidence of drug hypersensitivity reactions during such infections or autoimmune diseases. It implies that drug hypersensitivity reactions are actually caused by cross-reactivity of peptide-specific memory T cells, which happen to react also with some drugs.

In line with this notion is the observation that most drug-specific TCCs have been found to bear αβ TCRs, which usually recognize peptides, and that a general stimulation of T cells as in HIV or Epstein-Barr virus, cytomegalovirus infection, or exacerbation of autoimmune diseases is an important risk factor for drug hypersensitivity [37]. On the other hand, recent data suggest that the drug-induced T-cell activation leads to a reactivation of dormant herpes viruses and that the later appearing symptoms during drug hypersensitivity syndromes/drug rash with eosinophilia and systemic symptoms are largely related to reactivated herpes virus infections [38]. The incidence of SMX allergy in healthy subjects is ca. 2% to 4%, but during HIV infection, it might go up to ca. 50%, and amoxicillin hypersensitivity increases from 4% to 5% in healthy subjects to greater than 90% during an acute Epstein-Barr virus infection (infectious mononucleosis). Such immune reactions go along with the expansion of a polyclonal CD8^+ ^T-cell response, and such T cells are also found in the circulation of patients with maculopapular and bullous exanthema [39]. Even more, it seems likely that drug-specific cells exist even in individuals who are not hypersensitive. In an invitro study, several blood donors who had never been exposed to SMX nevertheless harbored SMX-and SMX-NO-specific cells in their T-cell repertoire [40].

### Clinical Data and Preferential Involvement of the Skin

Although the immune response to proteins, bacteria, viruses, and so on is well coordinated and localized to the affected organ, the immune response to a drug is often uncoordinated and generalized. It somehow circumvents the checkpoints for immune activation imposed by the classic antigen processing and presentation mechanisms, which may help to explain some of the peculiar pictures of drug hypersensitivity reactions such as:

a) the uncoordinated nature of drug allergic systemic reactions, which involve Th1, Th2, and CXCL 8-secreting T cells simultaneously;

b) the rapid appearance after the first encounter with the drug, without any sensitization phase;

c) some drugs causing delayed hypersensitivity reactions are not known to be metabolized to a chemically reactive compound; and

d) many chemically inert drugs, unable to form hapten-carrier complexes in the skin, are nevertheless able to cause positive skin tests with lymphocyte infiltration [39,41]. It is difficult to imagine that the locally applied drug in epicutaneous patch tests is transported to the liver, there metabolized and returns to the skin to cause a local reaction there.

Indeed, the p-i concept may better explain the generalized appearing skin symptoms than the hapten concept [42]. The p-i concept takes into account that drugs are ubiquitously distributed in the body, the lack of potent metabolism in the skin and the well-described sentinel function of some resident T cells in the skin [43]. Oral or parenteral uptake of drugs leads rapidly to a distribution throughout the body. This is particularly well documented for the skin, where for example, an antihistamine given orally can reach tissue levels within 45 to 60 minutes, which can block H1 receptors completely. This rapid distribution throughout the body may not only allow an interaction with an appropriate receptor but also with TCRs on certain T cells (p-i concept). Interestingly, the skin contains an extremely dense network of sentinel preactivated T cells (ready to react rapidly to an eventual "intruder") in close apposition to dendritic cells, which may predestine the skin to be the most frequently involved organ in drug hypersensitivity (based on the p-i concept). Such an environment provides ideal conditions for T-cell stimulation according to the p-i concept. Probably, these sentinel T cells may have a lower threshold of activation then naive T cells or circulating T cells because they are already preactivated [43].

### The p-i Concept and HLA Restriction in Drug Hypersensitivity

The p-i concept is not conceived as opposing the hapten or prohapten concept, but supplementing it. Certain drugs such as penicillins may cause hypersensitivity reactions caused by hapten-carrier formation. Others, such as quinolones and sulfanilamides, may cause hypersensitivity by the hapten and p-i concept simultaneously [4,22,24]. Thus, if the hapten concept is well proven for a certain drug such as *p*-phenylenediamine or SMX, it does not rule out that the p-i concept is not also playing some role.

The p-i concept is contradicting many well-established rules in immunology. However, this is no argument against it as it is no more immunology but pharmacology; and the findings in drug allergy do contradict many established rules [35]. Actually, the fact that the p-i concept is not implying an immune response to a drug may explain many of these puzzling findings. Drug hypersensitivity generated according to the p-i concept is not caused by a newly generated immune response to the drug but is the consequence of cross-reactivity. It does not require the involvement of the innate immune system to trigger immunity. Animal experiments aimed to prove or disprove the p-i concept by immunizing animals with the inert drug will necessarily fail-as inert drugs do not stimulate an immune response under normal conditions [44]. Actually, the p-i concept cannot be proven by usual immunologic tests but is better accessible to pharmacology of the TCR-drug interactions.

In the p-i concept, drugs are thought to primarily activate the TCR. How can one reconcile this concept with the striking HLA-B associations described for certain severe drug hypersensitivity reactions? Indeed, some of the most convincing HLA associations of disease were recently described for severe, often bullous, drug hypersensitivity reactions. For example, the abacavir-induced severe hypersensitivity reaction affecting multiple organs is strongly associated with the HLAB*5701 allele in whites,[45] the Stevens-Johnson syndrome after carbamazepine treatment in Han Chinese occurs in patients with the HLA-B*1502 allele,[33] and HLA-B*5801 is an important genetic risk factor for severe allopurinol-induced cutaneous adverse reactions such as Stevens-Johnson syndrome and toxic epidermal necrolysis [46]. It is clear that such strong associations with HLA alleles support an important role for HLA molecules in drug hypersensitivity. The HLA associations are usually explained by the presentation of certain peptides by a certain HLA allele. The hypothesis would be that certain peptides, presented rather exclusively by these HLA molecules, present the bound hapten in a particularly immunogenic form. However, albeit the association with HLA alleles is very strong, there are some open questions. Many patients with HLA-B*5801 are exposed to allopurinol, yet they do not develop hypersensitivity [46]. Whites do not show the association of HLA-B*1502 and carbamazepine hypersensitivity,[47] and most TCCs to carbamazepine generated from whites reacted with the parent compound [13]. Also and as reported by the authors of these studies, other factors located in this region of chromosome 6 may be important as well (eg, *HSP 70 *and other genes) [45,48]. Lastly, until now, no particular peptide or hapten-modified peptide could be identified and eluted from the HLA-B alleles [33].

**Figure 2:**
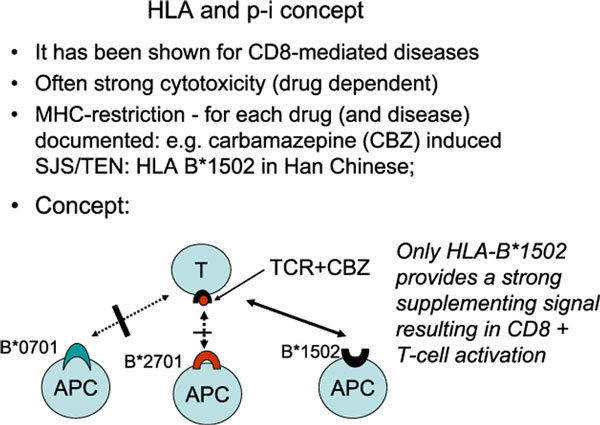
**HLA-allele association and p-i concept**. Some drugs elicit severe, often CD8-dominated, immune reactions in patients with a certain HLA-B alleles (carbamazepine and HLA-B*1502, allopurinol with HLA-B*5801, abacavir with HLA-B*5701). If these drugs stimulate via the p-i concept (as well documented for carbamazepine [CBZ], but not abacavir or allopurinol), then the strong HLA association would be explained by the ability of only the selected HLA allele to supplement the activation caused by the respective drug. In the example given, the HLA alleles B*0701 or B*2701 would be unable to supplement this initial signal by CBZ. However, none of the existing hypotheses can explain the finding that some of these alleles are found to be involved only in certain human races (eg, CBZ and HLA-B*1502 in Han Chinese). SJS indicates Stevens-Johnson syndrome; TEN, toxic epidermal necrolysis.

Thus, one could propose an alternative possibility. In the p-i concept, the drug binds primarily to a certain TCR. However, this binding needs supplementing interaction with an HLA molecule, which can only be given by a certain HLAB allele. In the absence of this HLA-B allele, the drugs cannot stimulate sufficiently (Figure 2).

## Conclusions

A series of clinical and laboratory investigations contradict the hapten model and suggest that the hapten model as the sole molecular explanation for drug-induced hypersensitivity may not be sufficient. We recently proposed the p-i concept, which supplements the hapten concept. In this concept, certain drugs are considered to be able to activate T cells by binding to T-cell receptors and subsequent cell activation. This explains many of the peculiar findings in drug hypersensitivity and opens new possibilities for immunopharmacology, as this drug binding may be stimulatory or inhibitory.
